# Intracellular Calcium Responses to External Calcium Stimuli in *Dictyostelium*

**DOI:** 10.4014/jmb.2412.12066

**Published:** 2025-04-27

**Authors:** Dahyeon Kim, Jiseong Seo, Taeck Joong Jeon

**Affiliations:** 1 Department of Integrative Biological Sciences & BK21 FOUR Educational Research Group for Age-Associated Disorder Control Technology, Chosun University, Gwangju 61452, Republic of Korea; 2 The Basic Science Institute of Chosun University, Chosun University, Gwangju 61452, Republic of Korea

**Keywords:** Calcium, G-proteins, cGMP, IplA, *Dictyostelium*

## Abstract

Intracellular calcium (Ca^2+^) plays a vital role in numerous cellular processes, including signal transduction, cell motility, and development. Despite extensive research on intracellular calcium dynamics, the specific mechanisms by which extracellular calcium influences intracellular calcium levels remain unclear. In this study, we generated a *Dictyostelium*-specific plasmid expressing the calcium biosensor GCaMP3 and visualized real-time intracellular calcium fluctuations in live cells. In response to external calcium, vegetative cells displayed a rapid and transient increase in intracellular calcium levels, peaking within 20 s, whereas aggregation-competent cells showed no such response. *Gβ*-null cells showed a fast and slight induction at approximately 5 s, with a slower and more efficient, extended high induction at 30 s. Meanwhile, *gca/sgc*-null cells showed a slightly delayed response, and a significantly lower proportion of *iplA*-null cells responded to external calcium stimuli compared to wild-type cells. In the presence of W7, a calmodulin antagonist that inhibits calcium release from acidic stores, cells exhibited a marked reduction in the major calcium induction peak at 20 s post-stimulation. Our results suggest that there are at least two pathways to increase the intracellular calcium level in response to external calcium stimuli, and that intracellular acidic calcium stores partially contribute to the second major peak of calcium induction following external stimuli. It appears that G proteins, cGMP, and IplA are involved in calcium homeostasis upon external calcium stimulation, and play an important role in modulating the timing and amplitude of calcium responses.

## Introduction

Calcium ions (Ca^2+^) are present in both the intracellular and extracellular environments of virtually all eukaryotic cells and play crucial roles in a wide array of cellular processes, including cell growth, cell death, cell motility, membrane fusion, cell-cell adhesion, and differentiation [1, 2]. Dysregulation of finely tuned calcium homeostasis has been linked to numerous pathologies, such as cancer, cardiovascular disease, bone disorders, and neurodegenerative diseases like Alzheimer's disease [2-4]. Understanding the regulation and dynamics of intracellular calcium concentration in response to extracellular stimuli is fundamental for elucidating these cellular processes.

*Dictyostelium discoideum*, a model organism renowned for its utility in studying cell motility, chemotaxis, multicellular development, and signal transduction, provides a unique system for exploring calcium dynamics across different developmental stages [5, 6]. Upon starvation, vegetative unicellular *Dictyostelium* cells migrate towards cAMP chemoattractants, which are periodically secreted by starving cells, and develop into a multicellular structure, finally forming a fruiting body [6, 7]. The development of *Dictyostelium* cells is significantly affected by both intracellular and extracellular calcium levels. In response to cAMP stimulation, cytosolic calcium levels transiently increase, and periodic oscillations in cytosolic calcium levels are observed during the aggregation stage of multicellular development [1, 7]. The increase in cytosolic calcium levels in response to the chemoattractant cAMP is mediated by multiple signaling pathways, including cAMP receptors, and both G protein-dependent and -independent pathways [1, 8, 9]. IplA, a *Dictyostelium* protein analogous to the mammalian IP_3_ receptor, is a ligand-gated calcium channel responsible for calcium release from the endoplasmic reticulum (ER)[10]. Calcium ATPases in the plasma membrane and acidic vesicles have also been suggested to contribute to cytosolic calcium oscillations and calcium elevation upon cAMP stimulation [1, 6, 11]. DdCAD-1 is a calcium-dependent extracellular adhesion molecule that contributes to multicellular development [6]. Additionally, cytosolic calcium elevation is induced by several external stimuli, including folate, differentiation-inducing factors DIF-1 and DIF-2, cyclic-di-GMP, L-glutamate, gamma-aminobutyric acid (GABA), and the polyketide MPBD (4-methyl-5-pentylbenzene-1,3-diol) [1, 6, 8, 12].

Calcium is crucial for cell motility and adhesion [1, 7]. Although calcium signaling is not strictly necessary for chemotaxis, cytosolic calcium levels influence cell migration by regulating the phosphorylation of myosin II heavy chains [13]. IplA and myosin heavy chain kinase have been reported to play important roles in the chemotactic responses to extracellular calcium gradients [10]. Cytosolic calcium levels are also involved in the regulation of cGMP signaling [9]. Extracellular calcium and calcium influx are important in establishing cell polarity during electrotaxis [14].

Despite numerous studies investigating the intracellular calcium responses to various stimuli, including chemical, electrical, and mechanical factors, the precise mechanisms by which extracellular calcium modulates intracellular calcium levels are not well characterized. In this study, we subcloned GCaMP3 into a *Dictyostelium* expression plasmid to establish a robust system for visualizing intracellular calcium changes. Using the GCaMP3 calcium biosensor, we monitored real-time calcium dynamics in *Dictyostelium*, focusing on the responses to external calcium stimuli. To elucidate the signaling molecules involved in this transduction, we investigated calcium responses in mutant cells lacking the G protein β subunit, IplA, and cGMP synthesis enzymes, as well as guanylyl cyclase and soluble guanylyl cyclase (GCA/sGC), and in the presence of inhibitors preventing the release of calcium out of intracellular stores.

## Materials and Methods

### Cell Culture

*Dictyostelium discoideum* wild-type KAx3 cells, *Gβ*-null cells (DBS0236531), *gca/sgcA*-null cells (DBS0236000), and *iplA*-null cells (DBS0266326) were obtained from the Dicty Stock Center (USA). *Dictyostelium* cells were cultured axenically in HL5 media or in association with *Klebsiella aerogenes* at 22°C. The knockout strains and transformants were maintained in 10 μg/ml blasticidin or 10 μg/ml G418. A calmodulin antagonist, W7, and 2,5-Di-tert-butylhydroquinone (BHQ) were purchased from MedChemExpress (USA). Dimethyl sulfoxide (DMSO) was purchased from Sigma-Aldrich (USA). Stock solutions of W7 (30 mM) and BHQ (200 mM) were prepared in DMSO, respectively. The calcium reporter plasmid in mammalian cells, pGCaMP3 [15], and a *Dictyostelium*-specific expression plasmid, pDXA-3C, were obtained from Addgene (USA) and Dicty Stock Center, respectively. Anti-green fluorescent protein (GFP) antibodies and horseradish peroxidase (HRP)-conjugated goat anti-mouse secondary antibodies were purchased from Santa Cruz Biotechnology (USA).

### Construction of a *Dictyostelium*-Specific Calcium Reporter Plasmid

A *Dictyostelium*-specific calcium reporter plasmid, pDXA-GCaMP3, was generated by subcloning the GCaMP region from pGCaMP3, a calcium reporter plasmid in mammalian cells [15] into the BamH1 site of the *Dictyostelium* expression vector pDXA-3C using a Gibson Assembly Cloning Kit (New England Biolabs, USA). The GCaMP region in pGCaMP3 plasmids was amplified by PCR using the pGCaMP3 plasmid as a template and a primer set (forward primer: 5’-GGTACCGAGCTCGCTCATCATCA-3’, reverse primer: 5’-CTAATGCATCTCGAGTGGCTCACTTCG-3’). The instructions provided by the kit manufacturer were followed. The plasmids were transformed into *Dictyostelium* cells by electroporation using a MicroPulser Electroporator (Bio-Rad, USA). Exponentially growing cells were washed and resuspended in ice-cold H-50 buffer (20 mM HEPES, 50 mM KCl, 10 mM NaCl, 1 mM MgSO_4_, 5 mM NaHCO_3_, and 1 mM NaH_2_PO_4_) at a density of 10^8^ cells/ml. Then, 1-10 mg of DNA was mixed with 100 μl of cell suspension in a prechilled cuvette and electroporated twice at 1 kV and 3 μF. The transformed cells were maintained in 10 μg/ml G418.

### Live-Cell Imaging and Analysis of Calcium Dynamics in GCaMP3-Expressing Cells

Vegetative *Dictyostelium* cells expressing GCaMP3 were grown in 6-well plates in HL5 media. Aggregation-competent cells were prepared by washing vegetative cells twice with development buffer (DB; 5 mM Na_2_HPO_4_, 5 mM KH_2_PO_4_, 2 mM MgCl_2_, and 1 mM CaCl_2_), resuspending them at a density of 5 × 10^6^ cells/ml, and then pulsing them with 30 nM cAMP at 6-min intervals for 5-6 h [16]. Vegetative or aggregation-competent cells were plated on glass-bottomed microwell plates and allowed to adhere for 30 min. The prepared cells were uniformly stimulated with 10 mM calcium. For uniform stimulation, a 100 mM calcium stock solution was prepared in DB buffer, and one-tenth volume of the stock solution was quickly and evenly added to the plates to reach a final concentration of 10 mM calcium. To assess the effects of W7 or BHQ inhibitors, the cells were incubated for an additional hour in the presence of 50 μM W7 or BHQ.

Fluorescence images were taken at time-lapse intervals of 2 s for 1 min using an inverted microscope (IX71; Olympus, Japan) equipped with a camera (DS-Fi1; Nikon, Japan) [17]. The frames were captured using NIS-Elements software (Nikon) and analyzed using ImageJ software (NIH, USA). Fluorescence intensities were measured with the ROI manager tool in ImageJ, and relative fluorescence intensities were calculated by dividing the intensity at each time point (Et) by the intensity before stimulation (Eo).

### Western Blot Analysis and Statistical Analysis

Collected cells were suspended in 0.1 ml sodium dodecyl sulfate (SDS) sample buffer (100 mM Tris-Cl [pH 6.8], 200 mM dithiothreitol, 4% SDS, 0.2% bromophenol blue, and 20% glycerol and heated at 95°C). Cells were then subjected to fluorescence with immunoblotting using anti-GFP antibodies.

Statistical significance was determined using a two-tailed *t*-test and *p*-values were calculated. As shown in Fig. 3C, a one-way ANOVA with a post-hoc Tukey HSD test was applied after checking the data normality with a Shapiro-Wilk test. The results of the normality test showed a normal distribution (*p*-values > 0.05 for all groups). All data were collected from at least three independent experiments and expressed as the means ± SD. A *p*-value of less than 0.05 was considered statistically significant.

## Results

### Live-Cell Imaging of Calcium Dynamics Using a Calcium Biosensor GCaMP3 in *Dictyostelium*

**Fig. 1:**
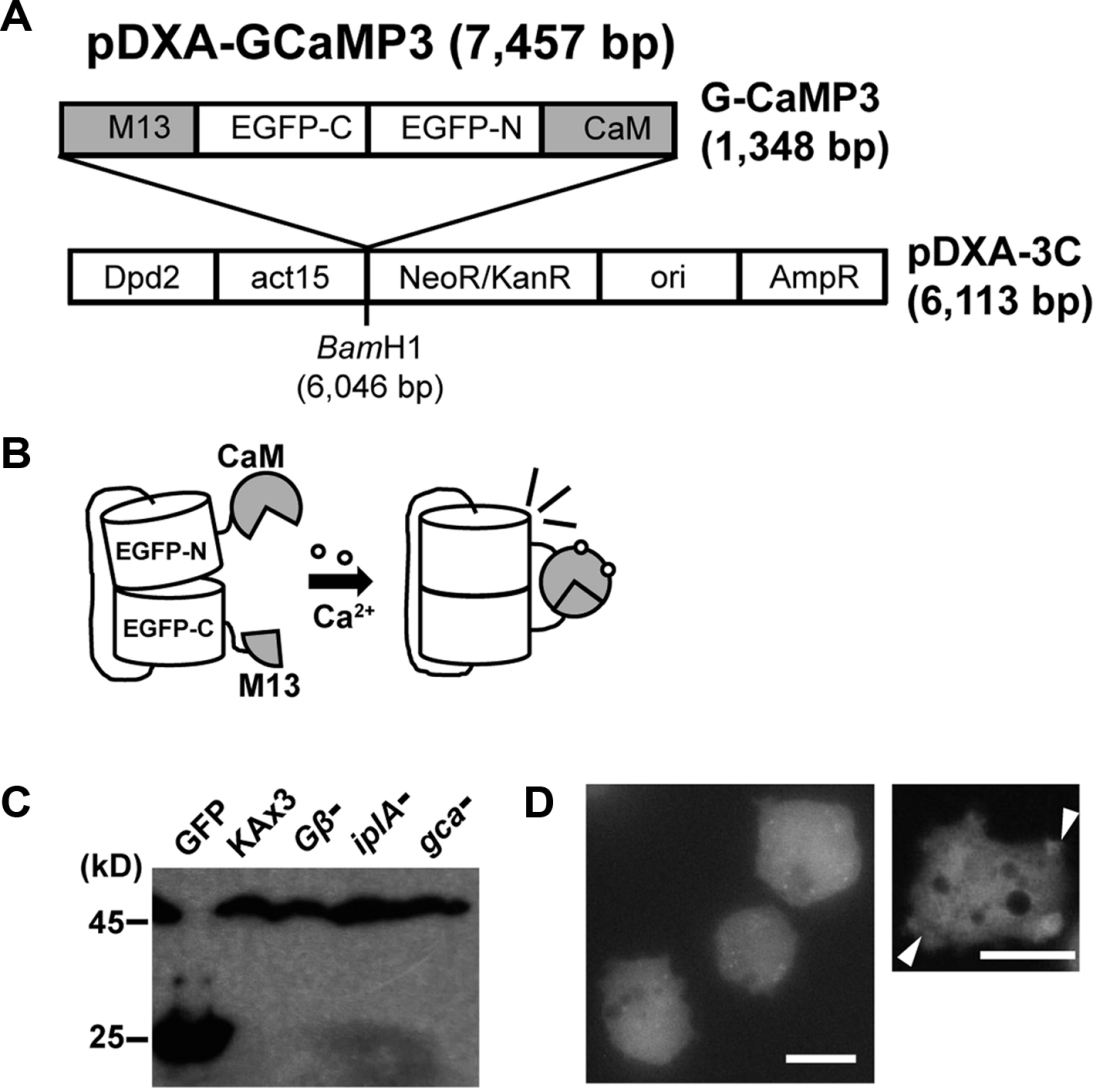
Schematic of a *Dictyostelium*-specific calcium reporter pDXA-GCaMP3 and its expression in *Dictyostelium* cells. (**A**) Design of pDXA-GCaMP3 plasmids. A *Dictyostelium*-specific calcium reporter plasmid, pDXA-GCaMP3, was developed by subcloning the GCaMP region (1,348 bp) from the mammalian expression plasmid pGCaMP3 (15) into the BamH1 site of the *Dictyostelium* expression vector pDXA-3C (6,113 bp). (**B**) Schematic of fluorescence expression upon calcium binding. GCaMP consists of a calcium-binding protein, calmodulin (CaM), a myosin light chain kinase (M13), and green fluorescent protein. Calcium-bound CaM interacts with M13, resulting in fluorescence. (**C**) Confirmation of GCaMP3 expression in *Dictyostelium* cells. The calcium reporter plasmid was introduced into wild-type (KAx3) and mutant cells. GCaMP3 expression was confirmed by immunoblotting with anti-GFP antibodies. Wild-type cells expressing empty GFP were used as a control in the first lane. (**D**) Fluorescence in vegetative *Dictyostelium* cells. Arrow heads indicate the protruding, ruffled regions of the cell. Scale bar = 5 μm.

In this study, a *Dictyostelium*-specific calcium sensor was developed to investigate the mechanism of intracellular calcium changes induced by external stimuli in *Dictyostelium*. The GCaMP portion, which is used as a calcium sensor plasmid in mammals [15], was subcloned into a *Dictyostelium*-expression plasmid and successfully expressed in *Dictyostelium*, allowing real-time visualization and quantification of calcium fluctuations within living cells (Fig. 1A and 1B). Originally developed for mammalian systems, GCaMP3 is a fusion of the calcium-binding protein calmodulin (CaM), a peptide sequence of myosin light chain kinase (M13), and a green fluorescent protein that fluoresces upon binding calcium [15]. The *Dictyostelium*-specific calcium sensor plasmid was introduced into wild-type KAx3 and mutant cells. The expression of the calcium sensor was confirmed by immunoblotting using anti-GFP antibodies, showing a band at approximately 45 kDa, which was the expected size of the calcium sensor fusion proteins, whereas empty GFP was detected at approximately 25 kDa (Fig. 1C). Fluorescence was observed within the cytoplasm, and intense fluorescence was shown in many granules within the cytoplasm (Fig. 1D). In some randomly ruffled cells, brighter fluorescence was observed at the ruffled edges.

### Intracellular Calcium Levels Transiently Increase in Response to External Calcium Stimuli

**Fig. 2:**
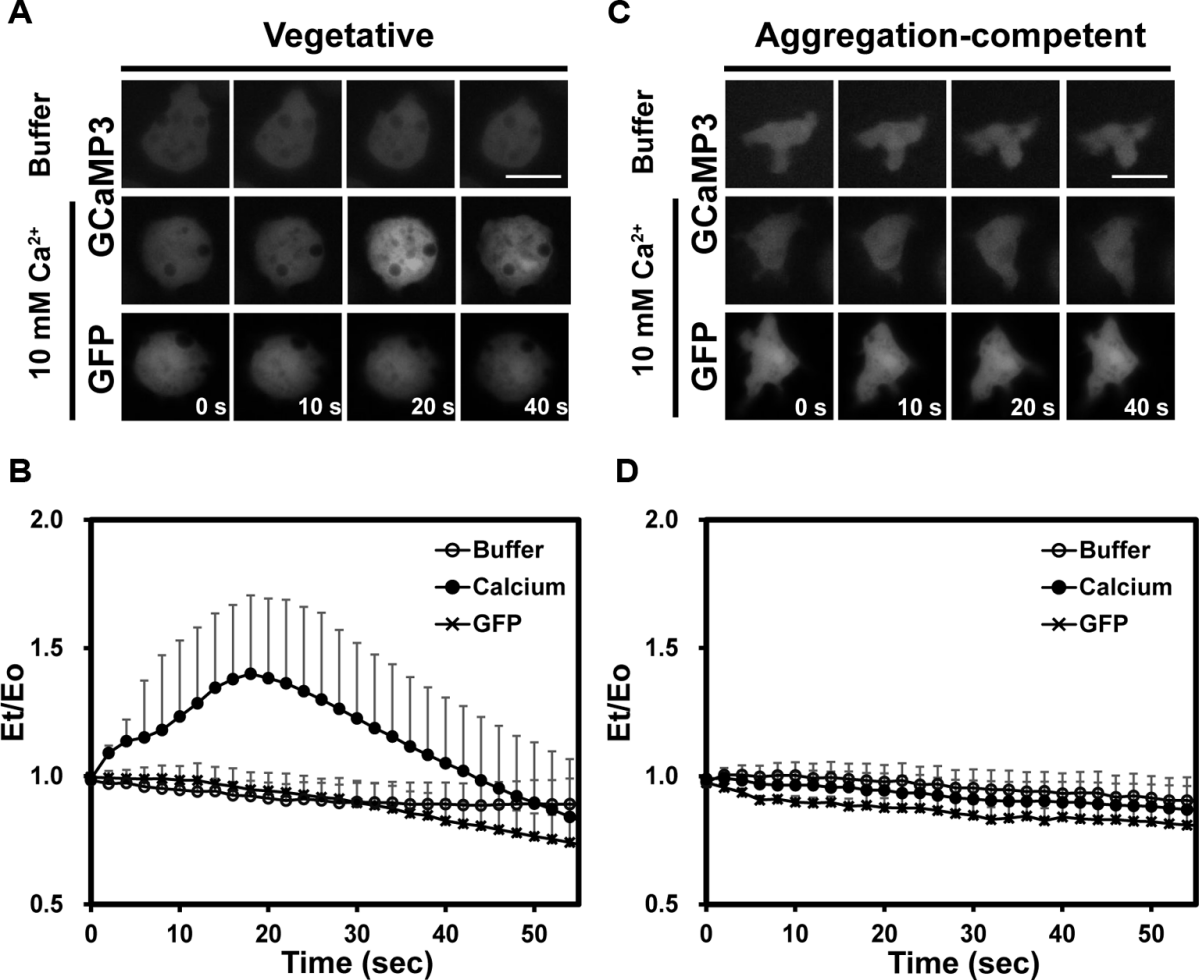
Intracellular [Ca^2+^]i response upon external calcium stimuli in wild-type cells. (**A**) [Ca^2+^]i response of vegetative wild-type cells. Vegetative wild-type cells expressing pDXA-GCaMP3 were uniformly stimulated with 10 mM calcium in DB buffer. Cells expressing empty GFP were used as a control. To determine if DB buffer alone induces fluorescence, DB buffer without 10 mM calcium was used as a negative control (first row). Fluorescence images were taken after stimulation, and representative images at the indicated time points are shown. Scale bar = 5 μm. (**B**) Dynamics of calcium levels in vegetative wild-type cells in response to external calcium stimulation. Fluorescence intensities were measured and quantified from timelapse recordings following calcium stimulation using ROI manager in ImageJ. The graphs represent the mean values from several cells, collected from at least three independent experiments. Error bars indicate SD. (**C**) [Ca^2+^]i response of aggregationcompetent wild-type cells. Aggregation-competent cells were prepared by pulsing the cells with cAMP at 6-min intervals for 6 h. Cells were then stimulated as described in panel A. (**D**) Dynamics of calcium levels in aggregation-competent cells following external calcium stimulation. Fluorescence intensities were quantified as described above.

First, to determine whether the calcium sensor responds to changes in calcium concentration, we examined intracellular calcium levels in response to external calcium stimuli in wild-type cells. Wild-type vegetative cells in DB buffer were rapidly and uniformly added with a high concentration of calcium up to a final concentration of 10 mM (Fig. 2A and 2B). Wild-type cells expressing empty GFP exhibited constant levels of fluorescence. When the cells were stimulated with buffers as a control, there was no change in the fluorescence. In contrast, when wild-type vegetative cells expressing GCaMP3 were stimulated with 10 mM calcium, fluorescence rapidly and transiently increased to 1.5-2 times the basal level, with a peak at approximately 20 s after stimulation, and then returned to the basal level within 40-50 s. These results indicate that intracellular calcium levels in vegetative cells transiently increase in response to external calcium stimuli and that the *Dictyostelium*-specific calcium reporter developed in this study sensitively responds to the intracellular calcium changes induced by external stimuli.

Upon starvation, *Dictyostelium* cells undergo a series of developmental progressions to finally form fruiting bodies. During development, both intra- and extracellular calcium concentrations fluctuate, influencing multicellular developmental processes [1, 6, 7]. To investigate the dynamics of intracellular calcium response to external calcium stimuli, we prepared aggregation-competent cells and examined the intracellular calcium response using a GCaMP3 calcium sensor. In contrast to vegetative cells, aggregation-competent cells developed over 6 h and expressing GCaMP3 exhibited no change in fluorescence intensity in response to either external calcium stimuli or DB buffer (Fig. 2C and 2D), and appeared the same as cells expressing empty GFP, showing a constant fluorescence intensity upon addition of external calcium. These data indicate no change in intracellular calcium levels in aggregation-competent cells, in contrast to vegetative cells, in response to external calcium stimuli. These findings suggest a development-dependent mechanism regulating the calcium response to external stimuli. Therefore, only vegetative cells were used in the subsequent experiments to determine the dynamic calcium response to external calcium stimuli.

### Multiple Signaling Pathways Are Involved in Calcium-Stimulated Intracellular Calcium Induction

Cytosolic calcium levels may increase via two main pathways: extracellular influx and intracellular calcium store release. The ER is primarily responsible for intracellular calcium storage [4, 9]. It has been previously reported that the intracellular calcium concentration is regulated in a G protein-dependent/-independent manner by the stimulation of external chemotactic factors, such as cAMP or folate [1, 8]. In addition, the phospholipase C (PLC) protein decomposes phosphatidylinositol 4,5-bisphosphate (PIP_2_), a phospholipid of the cell membrane, in response to external stimuli to create diacylglycerol (DAG) and inositol trisphosphate (IP_3_). IP_3_ is known to increase intracellular calcium concentration by releasing calcium accumulated in the ER into the cytoplasm [18]. cGMP is also known to play an important role in this process [8]. In this study, we examined if several signaling pathways including G proteins, IplA, and cGMP are involved in the process of intracellular calcium induction by external high concentration of calcium stimulation using cells lacking Gβ, GCA/sGC, or IplA.

**Fig. 3:**
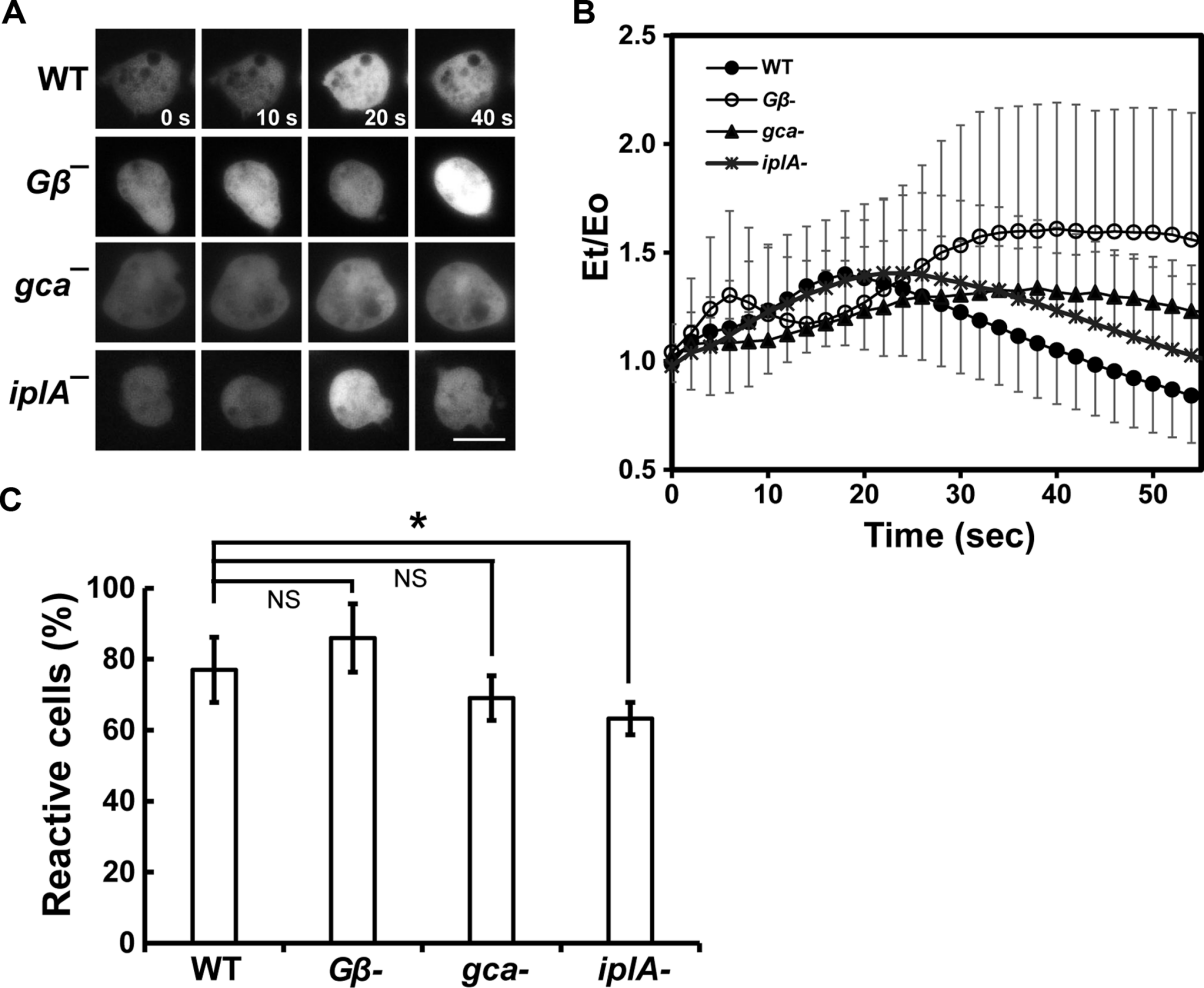
Intracellular [Ca^2+^]i response to external calcium stimuli in mutant cells. (**A**) [Ca^2+^]i response of mutants cells. Vegetative wild-type cells and mutant cells lacking Gβ, GCA/sGC, and IplA, all expressing pDXA-GCaMP3, were uniformly stimulated with 10 mM calcium. Fluorescence images were taken after stimulating the cells, and representative images at the indicated time points are presented. Scale bar = 5 μm. (**B**) Dynamics of calcium levels in vegetative mutant cells following external calcium stimulation. Fluorescence intensities of mutant cells were measured and quantified from time-lapse recordings using ROI manager in ImageJ. Graphs represent the mean values from at least three independent experiments. Error bars indicate SD. (**C**) Quantification of cells responsive to external calcium stimuli. The percentage of responsive cells was calculated from time-lapse recordings of calcium-stimulated cells expressing GCaMP3. The number of responsive cells was counted from the recordings and was divided by that of total cells. Statistical analysis was performed using one-way ANOVA followed by post-hoc Tukey HSD test. Statistically different from the control, *p < 0.05.

A *Gβ*-deficient cell line was used to investigate whether G proteins are involved in the induction of intracellular calcium increase by external calcium stimulation. *Gβ*-null vegetative cells showed a fast and slight induction at approximately 5 s, with a slower and more efficient, extended high induction at 30 s (Fig. 3A and 3B). Meanwhile, wild-type cells exhibited a major peak of induction at 20 s after external calcium stimulation. These results suggest that Gβ proteins are not essential for the increase of intracellular calcium concentration by external calcium but appear to be involved in the proper regulation of intracellular calcium levels upon external calcium changes through a yet-unknown mechanism.

Next, we investigated whether IplA and cGMP were related to an increase in calcium concentration using an *iplA*-deficient cell line and a *gca/sgc*-deficient cell line that cannot produce cGMP. *gca/sgc*-null cells showed slightly delayed responses to external calcium stimuli compared to wild-type cells (Fig. 3A and 3B). The maximum induction of intracellular calcium levels was observed approximately 30-40 s after stimulation, and the elevated intracellular calcium level extended to 60 s, suggesting that cGMP was likely involved in the recovery of calcium levels to the basal level after stimulation. *iplA*-null cells showed transient responses similar to those of wild-type cells, with a peak at 20 s (Fig. 3A and 3B). However, a significantly smaller proportion of cells responded to external calcium stimuli (Fig. 3C). While most of wild-type cells (approximately 80%) showed intracellular calcium induction, only approximately 60% of *iplA*-null cells were responsive to the external calcium stimuli. A slightly higher percentage of *Gβ*-null cells and a lower percentage of *gca/sgc*-null cells had a response to external calcium stimulation, but there was no significant difference compared to that of wild-type cells. These data suggest that IplA-related signaling pathways are involved in regulating intracellular calcium levels in response to external calcium stimuli.

### Acidic Calcium Storage Contributes to the Increase of Intracellular Calcium Level upon External Calcium Stimulation

An increase in the intracellular calcium concentration in response to external stimuli can be caused by the influx of extracellular calcium into the cell, or by the release of calcium into the cell from calcium storage sites, such as contractile vacuoles and the ER [1, 9, 11]. To determine whether intracellular calcium storage plays a role in increasing intracellular calcium levels upon external calcium stimulation, we examined calcium dynamics in the presence of calcium release inhibitors.

**Fig. 4:**
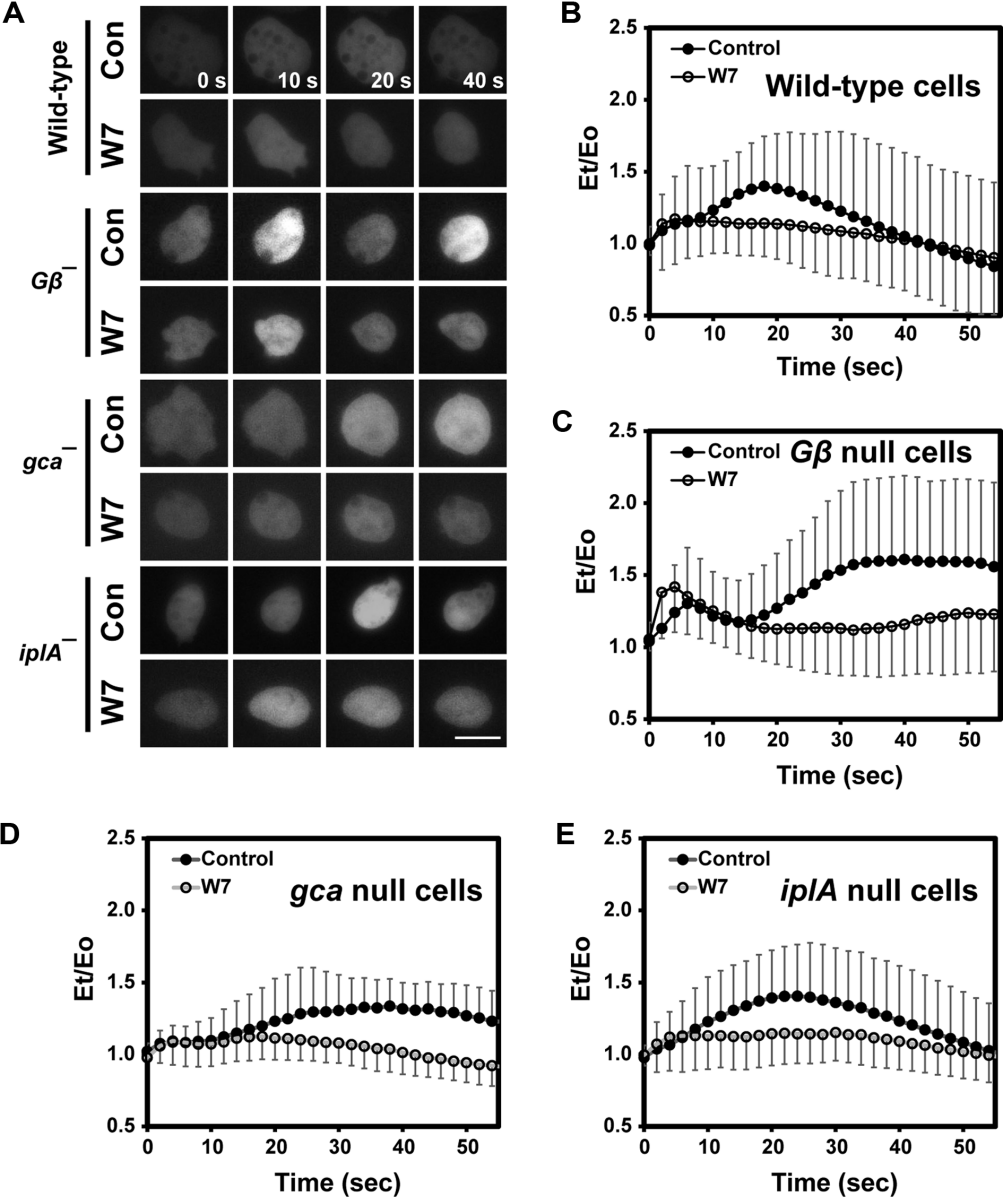
Intracellular [Ca^2+^]i response to external calcium stimuli in the presence of W7 inhibitors. (**A**) [Ca^2+^]i response of cells in the presence of W7 inhibitors. Wild-type and mutant cells expressing GCaMP3 were incubated with or without 50 μM W7 for at least 1 h and then stimulated with 10 mM external calcium. Fluorescence images were taken after stimulating the cells, and representative images at the indicated time points are presented. Scale bar = 5 μm. (**B**) Dynamics of calcium levels in wild-type cells in the presence of W7. Fluorescence intensities of wild-type cells in the presence of W7 were measured as described in Fig. 2 and compared with those of wild-type cells in the absence of W7 as a control. Error bars indicate SD. (**C**) Dynamics of calcium levels in mutants in the presence of W7. The fluorescence intensities of *Gβ*-null cells (**C**), gca/sgcnull cells (**D**), and *iplA*-null cells (**E**) in the presence of W7 were compared with their respective controls (absence of W7). Error bars indicate SD.

V-type H^+^ ATPases are present on the membranes of acidic calcium storage sites, such as contractile vacuoles, conferring a proton gradient to calcium uptake in the cytosol. Calmodulin is required for V-type H^+^ ATPase activity. W7, a calmodulin antagonist, prevents calcium from being released into cells from contractile vesicles [11, 19]. To determine whether contractile vesicles contribute to the observed calcium increase, cells were incubated in the presence of W7 and then treated with high concentrations of external calcium (Fig. 4). In the presence of W7, vegetative wild-type cells showed no such high transient induction of intracellular calcium levels 20 s after stimulation, as observed in control cells in the absence of W7, instead showing a fast and small induction at 3-5 s right after stimulation (Fig. 4A and 4B). The elevated calcium levels persisted over 50 s. The initial slight increase was not observed in the control cells in the absence of W7, but it was likely masked by the second major induction of intracellular calcium levels 20 s after stimulation. It seems that the initial slight induction in the presence of W7 appears to be revealed by blocking the major induction at 20 s. These results suggest that there might be two pathways to increase the intracellular calcium level in response to an external calcium stimulus, and that contractile vacuoles might partially contribute to the second major peak of calcium induction after stimulation.

To understand the roles of G proteins, cGMP, and IplA in the regulation of intracellular calcium levels, we examined the intracellular calcium responses of the mutant cells in the presence of W7. *Gβ*-, *gca/sgcA*-, and *iplA*-null cells showed a similar pattern of response, disappearance of the second major induction of intracellular calcium upon external calcium stimulation, as the wild type cells did in the presence of W7 (Fig. 4). In the absence of W7, *Gβ*-null cells showed the elevated first peak and the extended second peak of calcium induction compared to wild-type cells (Fig. 3). In contrast, *Gβ*-null cells, in the presence of W7, exhibited a complete disappearance of the second peak and a slightly increased and more apparent first peak compared to the control (Fig. 4C). Combined with the results of Fig. 3 which depicts *Gβ*-deficient cells, these results suggest there are at least two pathways in the induction of calcium level upon external calcium stimulation. It appears that G proteins might play a role in the initial increase of intracellular calcium levels upon external calcium stimulation, which is unlikely to be related to acidic calcium storage. Similarly, *gca/sgc*- and *iplA*-null cells in the presence of W7 showed no increase in intracellular calcium concentration upon external calcium stimulation (Fig. 4D and 4E). A slight increase initially after stimulation was observed in all mutants, as in wild-type cells, suggesting that the small increase immediately after external calcium treatment at 5 s is independent of G proteins, cGMP, IplA-involved pathways, and calcium release from acidic calcium storage.

BHQ acts as an inhibitor of sarcoendoplasmic reticulum (sER) calcium ATPase and is used to prevent calcium release from the sER [20, 21]. In the presence of 50 μM BHQ, no increase of intracellular calcium concentration was observed in response to external calcium stimulation in either wild-type or any mutant cell lines (Fig. S1). Distinctively, high fluorescence in cells in the presence of BHQ was observed even before calcium stimulation, indicating that the basal calcium level was highly elevated by the inhibition of sER calcium ATPase. Comparing intracellular calcium levels before stimulation, basal calcium levels in all strains were significantly increased in the presence of BHQ, but not in the presence of W7 (Fig. S2). In addition, *Gβ*-null cells had slightly higher basal levels compared to wild-type cells (Fig. S2). It has been reported that 100 μM BHQ increased cytoplasmic calcium concentration by more than 2.5-fold [22]. Moreover, it appears that the complete loss of intracellular calcium induction in the presence of BHQ upon external calcium stimulation is likely due to the highly elevated and saturated concentration of calcium at the basal level, rather than the blocking of calcium release from the ER. Further studies are required to determine whether the ER is responsible for the increase in intracellular calcium upon external calcium stimulation.

## Discussion

This study provides novel insights into the intracellular calcium dynamics in *Dictyostelium* in response to external calcium stimuli. Using the calcium biosensor GCaMP3, we successfully visualized and quantified intracellular calcium fluctuations in real time, demonstrating that calcium responses are developmentally regulated and influenced by multiple signaling pathways. Our findings contribute to a growing understanding of calcium homeostasis and signaling in *Dictyostelium*, building on prior knowledge of chemotactic and developmental calcium dynamics.

### External Calcium Stimuli Elicit Transient Calcium Elevation in Vegetative Cells

Our results showed that vegetative *Dictyostelium* cells exhibited a transient increase in intracellular calcium concentration upon exposure to 10 mM external calcium. This rapid calcium spike peaked at approximately 20 s and returned to baseline within 50 s, which is consistent with previous studies suggesting that extracellular calcium plays a pivotal role in intracellular signaling and cell motility [23]. However, our data diverge from those reported by Lusche *et al*. (2009) [23] in that our sensor system detected a more prolonged calcium response, which may be attributable to differences in the experimental conditions or sensor sensitivity. These findings confirm that intracellular calcium responses to external calcium are finely regulated and transient, supporting the notion that calcium homeostasis in *Dictyostelium* is tightly regulated [1, 6].

Importantly, aggregation-competent cells did not exhibit any changes in intracellular calcium levels upon external calcium stimulation, suggesting a developmental shift in calcium regulation. This observation aligns with previous reports indicating that calcium signaling is essential for cell polarity and movement during the vegetative phase but may play different roles during later developmental stages [8, 24]. The lack of response in aggregation-competent cells may reflect a switch in calcium utilization, where intracellular calcium dynamics are less influenced by external calcium stimuli during this phase. This developmental regulation of calcium signaling likely plays a crucial role in coordinating the multicellular organization of *Dictyostelium*.

### Role of G Proteins, IplA, and cGMP in Calcium Responses

Our analysis of the mutant strains provided further insights into the signaling pathways governing calcium homeostasis in *Dictyostelium*. The *Gβ*-null mutant, lacking the G protein β subunit, exhibited delayed and biphasic calcium responses to external calcium stimuli. This suggests that while G protein-coupled signaling is not essential for the initial calcium influx, it plays a significant role in modulating the timing and amplitude of calcium responses. This is consistent with previous studies demonstrating the involvement of G proteins in chemotactic calcium signaling via folate or cAMP in *Dictyostelium* [1, 8, 25].

Similarly, *iplA*-null cells deficient in the IP_3_ receptor homolog, which is responsible for calcium release from the ER, exhibited a significantly reduced population of responsive cells. These results underscore the importance of intracellular calcium release from stores for mediating a complete calcium response. Previous work by Berridge (2009) [26] established the pivotal role of IP_3_ receptors in regulating intracellular calcium stores, and our findings confirmed that IplA plays a critical role in *Dictyostelium*’s response to external calcium stimuli.

Our data also revealed that *gca/sgc*-null cells, which were deficient in cGMP synthesis, displayed delayed recovery to baseline calcium levels, suggesting that cGMP played a role in modulating post-stimulation calcium clearance. This is in line with the results of Schaap *et al*. (1996) [22], who identified a key role for cGMP in calcium signaling, particularly in facilitating the return to baseline calcium levels after a stimulus.

### Multiple Pathways Mediate Calcium Release from Intracellular Storage Sites

We further explored the contribution of intracellular calcium stores by employing W7, a calmodulin antagonist that inhibits calcium release from acidic storage sites such as contractile vacuoles [11, 19]. In the presence of W7, we observed a marked reduction in the major calcium induction peak at 20 s post-stimulation, suggesting that calcium release from contractile vacuoles significantly contributed to the overall calcium response. The presence of an unmasked initial calcium spike in W7-treated cells highlighted the existence of at least two distinct pathways governing intracellular calcium regulation, as supported by previous studies [11]. This dual-pathway model was further corroborated by our experiments using mutant strains in which both G protein-dependent and -independent mechanisms appeared to regulate calcium dynamics. Our findings with BHQ, an inhibitor of sER calcium ATPase [21], further suggest that the ER plays a crucial role in maintaining calcium homeostasis. Elevated basal calcium levels in BHQ-treated cells likely reflect calcium leakage from the ER, preventing additional calcium release upon stimulation. These results are consistent with the notion that sER calcium ATPase pumps are integral to intracellular calcium regulation [2, 4] and support the hypothesis that the ER is a major calcium storage site in *Dictyostelium*.

In conclusion, our study reveals that *Dictyostelium* employs a complex, developmentally regulated system to manage intracellular calcium levels in response to external stimuli. G proteins, IplA, cGMP, and intracellular stores contribute to the precise control of calcium homeostasis. Future research should focus on further dissecting the individual contributions of each pathway and identifying additional calcium-regulatory components, such as other calcium channels or pumps. Additionally, understanding the functional implications of calcium dynamics during *Dictyostelium* development and chemotaxis will provide further insights into the evolutionary conservation of calcium signaling mechanisms.

## Supplemental Materials

Supplementary data for this paper are available on-line only at http://jmb.or.kr.
