# Supplementary figures and images for: Intracellular Calcium Responses to External Calcium Stimuli in Dictyostelium
Source: J Microbiol Biotechnol. 2025 Apr 27;35:e2412066. doi: 10.4014/jmb.2412.12066 (PMC12089947; doi:10.4014/jmb.2412.12066)

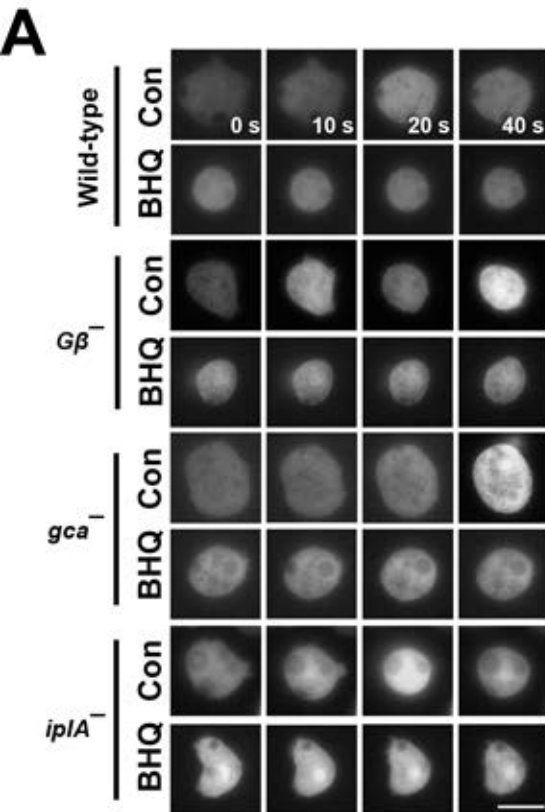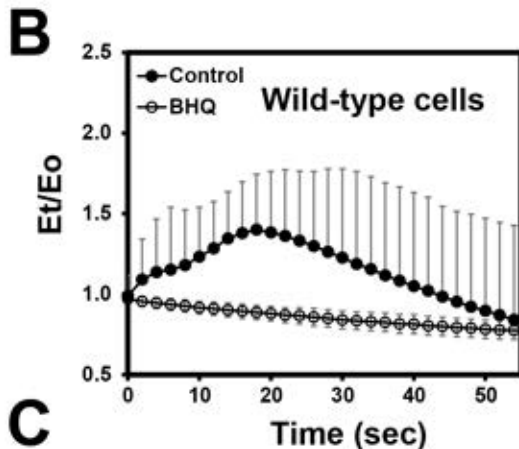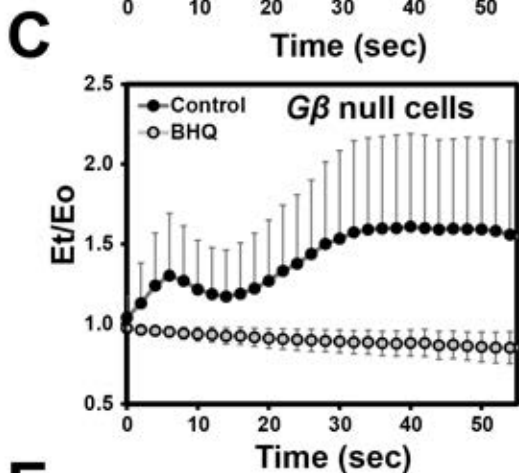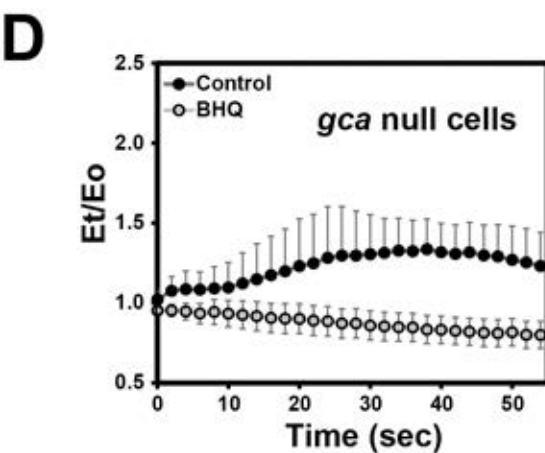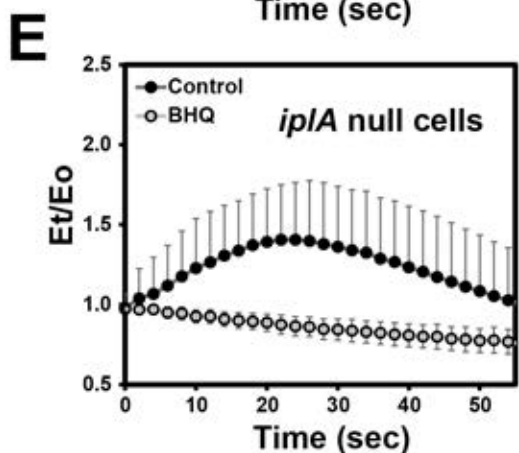

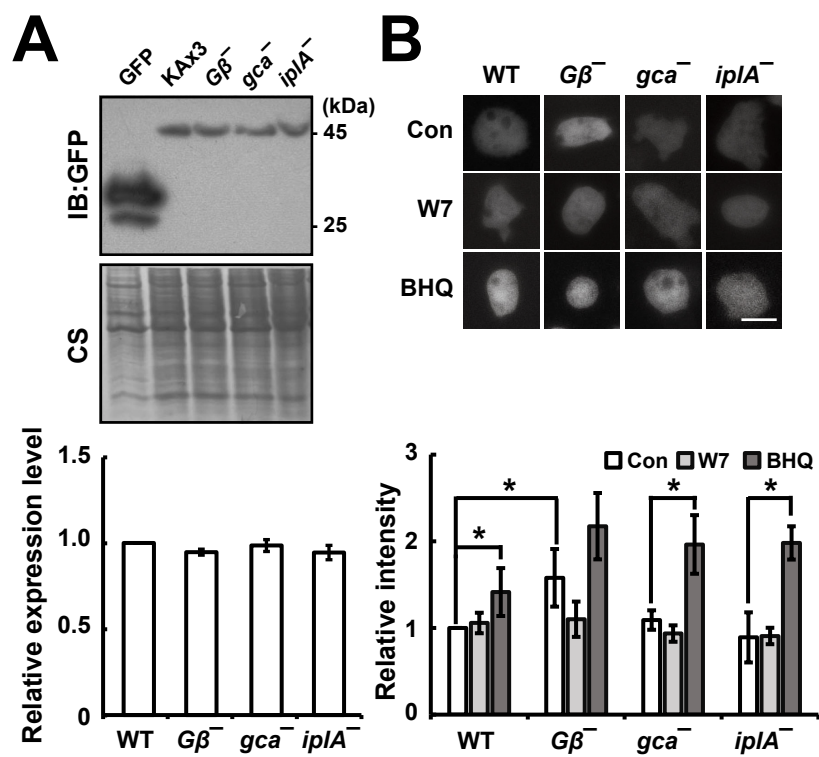

SuppFig2. Kim et al

Supplement: Supplementary file 1 [file jmb-35-e2412066-supple.pdf]
